# Association of Primary Care Clinic Appointment Time With Opioid Prescribing

**DOI:** 10.1001/jamanetworkopen.2019.10373

**Published:** 2019-08-30

**Authors:** Hannah T. Neprash, Michael L. Barnett

**Affiliations:** 1 Division of Health Policy and Management, School of Public Health, University of Minnesota, Minneapolis; 2 Department of Health Policy and Management, Harvard T.H. Chan School of Public Health, Boston, Massachusetts; 3 Division of General Internal Medicine and Primary Care, Department of Medicine, Brigham and Women’s Hospital, Boston, Massachusetts

## Abstract

**Importance:**

Time pressure to provide a quick fix is commonly cited as a reason why opioids are frequently prescribed in the United States, but there is little evidence of an association between appointment timing and clinical decision-making. As the workday progresses and appointments run behind schedule, physicians may be more likely to prescribe opioids.

**Objective:**

To estimate whether characteristics of appointment timing are associated with clinical decision-making about pain treatment.

**Design, Setting, and Participants:**

This cross-sectional study of physician behavior used data from electronic health record systems in primary care offices in the United States to analyze primary care appointments occurring in 2017 for patients with a new painful condition who had not received an opioid prescription within the past year.

**Main Outcomes and Measures:**

The association between treatment decisions and 2 dimensions of appointment timing (order of appointment occurrence and delay relative to scheduled start time) were assessed. The rates of opioid prescribing were measured and compared with rates of nonopioid pain medication (ie, nonsteroidal anti-inflammatory drugs) prescribing and referral to physical therapy. All rates were estimated within the same physician using physician fixed effects, adjusting for patient, appointment, and seasonal characteristics.

**Results:**

Among 678 319 primary care appointments (642 262 patients; 392 422 [61.1%] women) with 5603 primary care physicians, the likelihood that an appointment resulted in an opioid prescription increased by 33% as the workday progressed (1st to 3rd appointment, 4.0% [95% CI, 3.9%-4.1%] vs 19th to 21st appointment, 5.3% [95% CI. 5.1%-5.6%]; *P* < .001) and by 17% as appointments ran behind schedule (0-9 minutes late, 4.4% [95% CI, 4.3%-4.6%] vs ≥60 minutes late, 5.2% [95% CI, 5.0%-5.4%]; *P* < .001). Prescribing of nonsteroidal anti-inflammatory drugs and referral to physical therapy did not display similar patterns.

**Conclusions and Relevance:**

These findings suggest that, even within an individual physician’s schedule, clinical decision-making for opioid prescribing varies by the timing and lateness of appointments.

## Key Points

**Question:**

Is the decision to prescribe opioids associated with appointments that are behind schedule or later in the day compared with earlier or on-time appointments?

**Findings:**

In this cross-sectional study, opioid prescribing for opioid-naive patients with pain diagnoses was significantly associated with increases as the workday progressed and with appointments that started late, although the effect size was modest. Nonopioid pain treatment orders did not show similar patterns.

**Meaning:**

Appointment timing that contributes to time pressure could be adversely associated with physician decision-making and could have widespread relevance for public health and quality improvement efforts, if similar patterns exist in other clinical scenarios.

## Teaser

This cross-sectional study of physician behavior examines whether the order and lateness of primary care appointments is associated with opioid prescribing.

## Introduction

There is growing recognition that the increasing rate of opioid prescribing in the past 3 decades has been a major contributor to the national crisis of opioid use disorder and overdose.^1^ The volume of opioids prescribed in the United States nearly tripled from the early 1990s to their peak in 2011.^2^ This increase was accompanied by an approximately 4-fold increase from 1999 to 2016 in the rate of opioid overdose deaths, with more than 42 000 deaths in 2016 alone.^3^ In recent years, incident opioid prescribing has decreased, although many physicians continue to write high-risk opioid prescriptions.^4^

Many observers have blamed chaotic practice environments (ie, increasing financial pressure, productivity expectations, and the cognitive effort of caring for complex patient populations) for high rates of opioid prescribing because opioids can be a quick fix for a visit where pain is a symptom.^5,6,7,8^ One author frankly described the tension between maintaining high clinical volume vs having time-consuming discussions: “Many physicians may behave in a way even they think is questionable: write the requested opioid prescription, and move on.”^5^ The concept that time pressure can drive physician decision-making is long-standing, but little empirical literature has examined the existence of this phenomenon or its magnitude.^9^

Existing evidence suggests that appointment timing could play an important role in physicians’ prescribing decisions.^10,11^ For example, one study^10^ found that rates of inappropriate antibiotic prescribing increased over the course of the workday, supporting the idea that physicians’ ability to perform effortful clinical tasks, such as counseling against antibiotic prescribing, can degrade over time. Similarly, avoiding opioid prescribing risks disappointing patients and making visits longer, because time is needed to explain nonopioid alternatives for pain management. However, defaulting to an opioid prescription has clinical ramifications because even short, self-limited prescriptions of opioids can play a substantial role in patients transitioning to long-term opioid use.^12,13,14,15^

We hypothesized that as the workday progresses and appointments run behind schedule, physicians may be more likely to prescribe opioids in potentially inappropriate situations to simply move on. Using a national sample of all-payer claims and electronic health record (EHR) data, we examined within-physician changes in opioid prescribing behavior over the course of the day compared with other nonopioid forms of pain treatment, such as nonsteroidal anti-inflammatory drugs (NSAIDs) and physical therapy referrals.

## Methods

### Study Population

We used a subset of claims and EHR data from athenahealth Inc, a cloud-based health care information technology company that provides physician practices with medical billing, practice management, and EHR services. These data have been used in prior work^11,16,17,18,19^ to assess trends in opioid and antibiotic prescribing.

Our study sample included primary care appointments for adult patients with a new painful condition and no observable long-term use of opioids. To define this sample, we began with the universe of outpatient office appointments (during 2017) to physicians with a primary specialty of internal medicine, family practice, or general practice. We identified appointments that recorded a painful diagnosis, using the Centers for Disease Control and Prevention’s classification of diagnosis codes for chronic pain^20^ and grouping them into 5 distinct clinical categories: back pain, headache, joint disease, other musculoskeletal conditions including fibromyalgia, and other pain syndromes (eTable 1 in the Supplement). We further categorized these pain diagnoses as new or existing, on the basis of the presence of a within-category diagnosis within the patient’s prior 12 months of claims, and retained only appointments where a new painful diagnosis was recorded. To exclude patients currently receiving opioid therapy, we restricted our sample to patients who had not received a prescription for an opioid from any clinician in our data set within the past year. We also excluded patients with a current cancer diagnosis.

We made additional sample restrictions to standardize the appointment types and working conditions for physicians in our sample. We included only appointments for established patients (ie, not new to the practice), scheduled for the most common appointment durations of 10, 15, 20, or 30 minutes and scheduled more than 48 hours in advance. We further restricted the sample to appointments that occurred on a weekday (ie, Monday through Friday), during which the physician conducted at least 10 appointments. To ensure adequate sample size, we excluded primary care physicians with fewer than 40 appointments meeting our criteria. Complete sample selection criteria are provided in the eAppendix in the Supplement.

This study was approved by the institutional review board at the University of Minnesota, which determined it not to be human subjects research, as defined by US Department of Health and Human Services and US Food and Drug Administration regulations (University of Minnesota worksheet HRP-310). The need for informed consent was waived because the data were deidentified. This study follows the Strengthening the Reporting of Observational Studies in Epidemiology (STROBE) reporting guideline.

### Appointment Timing

We used EHR scheduling information to calculate each appointment’s start time and ranked appointments according to the order in which they occurred within the workday. We examined appointment order in groups of 3 (chosen to balance having adequate sample size within each group with the smallest grouping possible) up to 21 appointments for estimating 95% confidence intervals. Less than 5% of the appointments in our sample were the 22nd appointment of the day or later.

We calculated an appointment’s lateness by comparing the EHR time stamp for a physician starting an encounter with a patient with that appointment’s scheduled start time. We categorized appointment lateness in 10-minute increments (0-9, 10-19, 20-29, 30-39, 40-49, and 50-59 minutes) or at least 60 minutes late.

### Outcomes and Patient Covariates

Our primary outcome of interest was opioid prescription ordered by the primary care physician, defined as a binary indicator equal to 1 when there was an opioid prescription linked to an appointment (eAppendix and eTable 2 in the Supplement) by exact patient identifier, physician identifier, and date. Because we did not have pharmacy data, we could not observe whether the prescription was filled, so our outcome should be interpreted as the physician’s intention for the patient to receive opioids. We examined a secondary outcome for nonopioid pain management that we hypothesized would be less likely to conform to patient expectations: prescribing of NSAIDs (eTable 2 in the Supplement). We also examined the outcome of referral to a physical therapist, a nonmedication therapy that is delayed and will not treat pain immediately. From submitted service claims, we collected information including patient’s age, sex, primary insurer (eg, commercial, Medicare, Medicaid, or uninsured), pain category and status (eg, new or existing pain), and chronic condition count (see details in eAppendix in the Supplement).

### Statistical Analysis

We used multivariable linear probability models to assess the association between appointment time and prescribing for opioid-naive patients with a painful condition. Dependent variables were the occurrence of primary or secondary outcomes. The key explanatory variable was a categorical variable indicating appointment timing (eg, appointment order or lateness). We controlled for the aforementioned patient characteristics, scheduled appointment duration, and the quarter-year (eg, January to March 2017) during which the appointment occurred to control for secular time trends. Recognizing that lateness typically accumulates over the course of a physician’s day (ie, the last appointment is more likely to start late than the first), analyses of lateness as the exposure of interest also included indicators for appointment start hour. By accounting for expected changes over the course of the day, this allowed us to isolate within-hour effects of a delayed appointment start. Our main analysis models included physician fixed effects to capture time-invariant observable and unobservable (eg, practice style) differences across physicians that may be associated with opioid prescribing behaviors. With physician fixed effects, our estimates represent average within-physician estimates of the association between prescribing and appointment timing.

We used Huber-White robust variance estimators in all models to account for correlated data within physicians. All analyses were performed with the use of Stata statistical software version 15.1 (StataCorp). All 95% confidence intervals reflect 0.025 in each tail, or 2-tailed *P* ≤ .05.

We also estimated the change in the total number of opioid prescriptions in our sample if the timing for the earliest set of appointments had held constant. To do so, we multiplied the adjusted prescribing rate for each appointment rank by the number of appointments at that rank in our sample and then summed across ranks to calculate the adjusted total number of opioids prescribed with the current distribution of appointment timing. From this, we subtracted the total number of opioids that would have been prescribed if the prescribing rate for the first 3 appointments of the day held constant over time.

### Sensitivity Analyses and Falsification Tests

We performed sensitivity analyses to assess the potential role of selection bias in our analysis. We replicated our main analysis, restricting the sample to appointments with observably similar patient and appointment characteristics. We first restricted by payer type, examining the Medicare and commercially insured populations separately. We further restricted within payer by scheduled duration, including only the most common appointment duration (15 minutes). As an additional robustness test, we estimated a multivariable logistic regression with physician random effects to compute adjusted odds ratios (eAppendix in the Supplement).

We included 2 falsification tests, examining rates of initial (ie, first prescription within the past year) antihypertensive and statin prescribing (eAppendix in the Supplement). Because these medications are prescribed for nonacute, asymptomatic conditions, we expected to see either no association or an inverse association between prescribing rates and appointment timing.

## Results

Our sample consisted of 678 319 appointments for 642 262 opioid-naive patients (392 422 [61.1%] women) with painful conditions seeing 5603 primary care physicians during 2017. Compared with a national sample of office-based appointments for pain-related conditions, the patients in our sample were older and more likely to have Medicare insurance (eTable 3 in the Supplement).

Several patient and appointment characteristics had statistically significant differences by appointment order (Table 1). For example, patients seen during the first 6 appointments of the day were less likely to be female than patients seen in the 13th through 21st appointments occurring later in the day (58.6% vs 62.5%; difference in means, 3.9% [95% CI, 3.7%-4.3%]; *P* < .001), less likely to have no chronic conditions (30.1% vs 37.2%; difference in means, 7.1% [95% CI, 6.8%-7.4%]; *P* < .001), and less likely to have a short (≤15 minutes) appointment (75.2% vs 79.9%; difference in means, 4.6% [95% CI, 4.4%-4.9%]; *P* < .001). Overall, opioids were prescribed in 4.7% of the appointments in our sample; prescription rates varied by patient characteristics (eTable 4 in the Supplement) and across individual primary care physicians (eFigure 1 in the Supplement).

**Table 1.** Patient and Appointment Characteristics, by Appointment Order

Characteristic	Appointment Order, No. (%)^a^		
	1st to 6th (n = 274 408)	7th to 12th (n = 227 211)	13th to 21st (n = 176 700)
Age category, y			
25-44	37 315 (13.6)	32 002 (14.1)	28 999 (16.4)
45-64	116 785 (42.6)	89 146 (39.2)	76 806 (43.5)
≥65	120 308 (43.8)	106 063 (46.7)	70 895 (40.1)
Female	160 672 (58.6)	145 374 (64.0)	110 504 (62.5)
Insurance status			
Commercial	131 986 (48.1)	96 371 (42.4)	86 483 (48.9)
Medicare	111 354 (40.6)	99 434 (43.8)	66 231 (37.5)
Medicaid	15 332 (5.6)	15 310 (6.7)	11 648 (6.6)
Medicare plus Medicaid	10 463 (3.8)	11 356 (5.0)	8267 (4.7)
Uninsured	3544 (1.3)	3028 (1.3)	2647 (1.5)
Other payer	1729 (0.6)	1712 (0.8)	1424 (0.8)
Pain category^b^			
Back	84 666 (30.9)	72 660 (32.0)	56 861 (32.2)
Joint	133 991 (48.8)	109 433 (48.2)	82 823 (46.9)
Musculoskeletal	76 131 (27.7)	63 243 (27.8)	50 233 (28.4)
Migraine	17 447 (6.4)	14 053 (6.2)	11 648 (6.6)
Other	11 812 (4.3)	12 027 (5.3)	9322 (5.3)
Chronic condition			
0	82 564 (30.1)	76 643 (33.7)	65 706 (37.2)
1	74 972 (27.3)	62 134 (27.3)	48 603 (27.5)
≥2	116 872 (42.6)	88 434 (38.9)	62 391 (35.3)
Scheduled duration, min			
10	18 556 (6.8)	15 852 (7.0)	14 168 (8.0)
15	187 929 (68.5)	156 913 (69.1)	126 983 (71.9)
20	36 898 (13.4)	31 023 (13.7)	21 335 (12.1)
30	31 025 (11.3)	23 423 (10.3)	14 214 (8.0)

^a^ Appointment order was determined on the basis of the observed start time of each appointment within each physician’s workday. *P* values from 1-way analyses of variance are less than .01.

^b^ Percentages may not sum to 100, because patients presented with multiple types of pain.

### Appointment Timing and Opioid Prescribing

We observed increasing rates of opioid prescribing as appointments progressed through the day and as they ran behind schedule (Figure). Controlling for patient characteristics, appointment characteristics, and physician fixed effects, compared with the 1st to 3rd appointments, the 19th to 21st appointments were 1.3 percentage points more likely to result in an opioid prescription (5.3% [95% CI, 5.1%-5.6%] vs 4.0% [95% CI, 3.9%-4.1%]; *P* < .001) (eTable 5 in the Supplement shows full regression results), representing a 33% relative increase in opioid prescribing over the day. The association between appointment order and increasing opioid prescribing rate was robust to multiple sensitivity analyses, including sequential sample restrictions to eliminate systematic variation in payer or scheduled appointment duration (Table 2) and the use of multivariable logistic regression to compute adjusted odds ratios (eFigure 2 in the Supplement). Within our sample of 678 319 appointments, if the opioid prescribing rate for the first 3 visits had held constant throughout the day, there would have been 4459 fewer opioid prescriptions in 2017.

**Figure.**
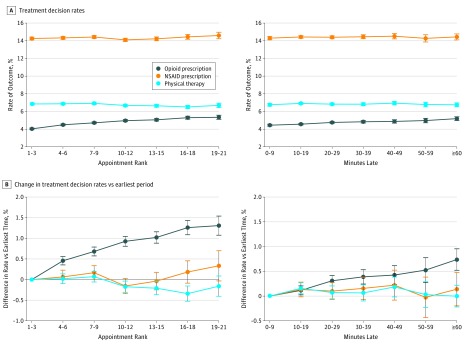
Pain Treatment Decision Rates and Changes in Rates by Appointment Timing Graphs show the absolute rate of each treatment decision by appointment rank and lateness (A) and the absolute percentage point change vs the earliest period (1st to 3rd appointments, or 0-9 minutes late) (B). Point estimates (circles) and 95% confidence intervals (whiskers) were estimated with the use of multivariate linear regression models with opioid prescribing, nonsteroidal anti-inflammatory drug (NSAID) prescribing, and referral to physical therapy as the dependent variables. The key covariate was appointment order and appointment lateness, respectively. All models were adjusted for patient characteristics (age category, sex, insurer, chronic condition count, and pain category), scheduled appointment duration, season-year fixed effects, and physician fixed effects. When appointment lateness was the key covariate, models were additionally adjusted for appointment start hour.

**Table 2.** Adjusted Rates of Opioid Prescribing by Appointment Order, Main Estimate and Subsamples

Appointment Order	Rate of Opioid Prescribing, % (95% CI)^a^				
	Main Model (N = 678 319)	Appointments			
		Only Medicare (n = 277 019)	Only 15-min Medicare (n = 193 882)	Only Commercial (n = 314 840)	Only 15-min Commercial (n = 217 772)
1st-3rd	4.03 (3.94-4.13)	4.43 (4.27-4.59)	4.56 (4.37-4.76)	3.10 (2.98-3.21)	3.16 (3.02-3.30)
4th-6th	4.49 (4.39-4.59)^b^	4.84 (4.69-4.99)^b^	4.98 (4.80-5.17)^b^	3.64 (3.50-3.78)^b^	3.77 (3.59-3.94)^b^
7th-9th	4.72 (4.61-4.82)^b^	5.24 (5.07-5.41)^b^	5.19 (4.99-5.40)^b^	3.74 (3.59-3.89)^b^	3.87 (3.69-4.06)^b^
10th-12th	4.96 (4.84-5.08)^b^	5.45 (5.26-5.64)^b^	5.50(5.27-5.73)^b^	4.06 (3.90-4.23)^b^	4.20 (4.00-4.41)^b^
13th-15th	5.06(4.92-5.20)^b^	5.50 (5.26-5.74)^b^	5.49 (5.22-5.77)^b^	4.04 (3.85-4.22)^b^	4.11 (3.88-4.34)^b^
16th-18th	5.29 (5.12-5.46)^b^	6.02 (5.71-6.34)^b^	6.09 (5.71-6.47)^b^	4.25 (4.03-4.47)^b^	4.38 (4.12-4.65)^b^
19th-21st	5.34 (5.11-5.57)^b^	5.94 (5.53-6.35)^b^	5.93 (5.46-6.41)^b^	4.20 (3.91-4.48)^b^	4.21 (3.88-4.54)^b^

^a^ Column 1 presents results from the main specification, identical to data shown in the Figure. Subsequent columns present results from the subsample analyses, designed to reduce patient heterogeneity over the course of the day. Column 2 includes only appointments for Medicare beneficiaries. Column 3 includes only 15-minute appointments for Medicare beneficiaries. Column 4 includes only appointments for commercially insured individuals. Column 5 includes only 15-minute appointments for commercially insured individuals. All models were adjusted as described in the Figure.

^b^ *P* < .01 vs first to third appointments.

Comparing appointments by lateness (ie, observed start time relative to scheduled start time), patient and appointment characteristics had small, statistically significant differences (eTable 6 in the Supplement). Controlling for appointment start hour, opioid prescribing increased as appointments started later, from 4.4% (95% CI, 4.3%-4.6%) for appointments starting 0 to 9 minutes late to 5.2% (95% CI, 5.0%-5.4%) for appointments 60 minutes or more late (*P* < .001 vs 0-9 minutes late), a 17% relative increase (Figure) (eTable 7 in the Supplement shows full regression results). The association between appointment lateness and increasing opioid prescribing rate was robust to multiple sensitivity analyses, including sequential sample restrictions to eliminate systematic variation in payer or scheduled appointment duration (Table 3) and the use of multivariable logistic regression to compute adjusted odds ratios (eFigure 3 in the Supplement).

**Table 3.** Adjusted Rates of Opioid Prescribing by Appointment Lateness, Main Estimate and Subsamples

Minutes Late	Rate of Opioid Prescribing, % (95% CI)^a^				
	Main Model (n = 593 819)	Appointments			
		Only Medicare (n = 239 096)	Only 15-min Medicare (n = 166 992)	Only Commercial (n = 279 576)	Only 15-min Commercial (n = 193 234)
0-9	4.45 (4.33-4.56)	4.82 (4.62-5.01)	4.97 (4.74-5.20)	3.52 (3.37-3.67)	3.62 (3.43-3.80)
10-19	4.56 (4.47-4.65)^b^	5.10 (4.95-5.26)^c^	5.18 (4.99-5.36)	3.58 (3.47-3.70)	3.72 (3.58-3.86)
20-29	4.76 (4.65-4.86)^b^	5.30 (5.12-5.48)^b^	5.31 (5.09-5.52)^d^	3.75 (3.61-3.89)^c^	3.88 (3.71-4.06)
30-39	4.83 (4.69-4.98)^b^	5.42 (5.17-5.66)^b^	5.47 (5.18-5.77)^c^	3.87 (3.68-4.05)^b^	3.99 (3.76-4.22)^c^
40-49	4.87 (4.68-5.06)^b^	5.33 (5.02-5.65)^c^	5.32 (4.94-5.70)	3.98 (3.72-4.24)^b^	4.05 (3.73-4.37)^c^
50-59	4.97 (4.72-5.22)^b^	5.60 (5.17-6.02)^b^	5.90 (5.38-6.41)^b^	3.75 (3.41-4.09)	3.79 (3.38-4.20)
≥60	5.18 (4.97-5.40)^b^	5.54 (5.19-5.88)^b^	5.58 (5.16-6.00)^c^	4.40 (4.08-4.72)^b^	4.53 (4.14-4.91)^b^

^a^ Column 1 presents results from the main specification, identical to data shown in the Figure. Subsequent columns present results from the subsample analyses, designed to reduce patient heterogeneity over the course of the day. Column 2 includes only appointments for Medicare beneficiaries. Column 3 includes only 15-minute appointments for Medicare beneficiaries. Column 4 includes only appointments for commercially insured individuals. Column 5 includes only 15-minute appointments for commercially insured individuals. All models were adjusted as described in the Figure.

^b^ *P* < .01 vs appointments that started ahead of schedule.

^c^ *P* < .05 vs appointments that started ahead of schedule.

^d^ *P* < .10 vs appointments that started ahead of schedule.

### Appointment Timing and Other Ordering Behavior

Among the same sample of appointments, we did not observe a similar association between appointment timing and NSAID prescribing or referrals for physical therapy (Figure). Overall, NSAIDs were prescribed in 14.3% of appointments, with little change as the day progressed (1st to 3rd appointments, 14.3% [95% CI, 14.1%-14.4%] vs 19th to 21st appointments, 14.6% [95% CI, 14.2%-15.0%]; *P* = .12) or as appointments started behind schedule (0-9 minutes late, 14.3% [95% CI, 14.1%-14.5%] vs ≥60 minutes late, 14.4% [95% CI, 14.1%-14.8%]; *P* = .52).

A referral to physical therapy was ordered during 6.8% of appointments in our sample, also without a statistically significant increase as the day progressed (1st to 3rd appointments, 6.8% [95% CI, 6.7%-7.0%] vs 19th to 21st appointments, 6.7% [95% CI, 6.4%-6.9%]; *P* = .28), or as appointments started behind schedule (0-9 minutes late, 6.8% [95% CI, 6.6%-6.9%] vs ≥60 minutes late, 6.8% [95% CI, 6.5%-7.0%]; *P* = .98). Non–pain-related prescribing behavior, including initial orders for antihypertensives and statins, did not increase as a function of appointment order (eFigure 4 in the Supplement) or appointment lateness (eFigure 5 in the Supplement).

## Discussion

In a large, national sample of primary care appointments, we observed within-physician variation in opioid prescribing associated with the timing of an appointment. Physicians were significantly more likely to prescribe opioids as the workday progressed and as appointments started later than scheduled. As already noted, within our sample of appointments, if the opioid prescribing rate for the first 3 visits had held constant throughout the day, there would have been 4459 fewer opioid prescriptions in 2017. Although the absolute difference in prescribing rate across the day of 1.3 percentage points is modest, it is similar in magnitude to the 0.9% absolute reduction in monthly incidence of initial opioid prescribing that occurred nationwide from 2012 to 2017,^4^ a period when overall opioid prescription volume decreased substantially in the United States. This finding suggests that a change in prescribing behavior of this magnitude could have meaningful relevance for national trends in opioid use. However, the associations we observe are relatively small compared with the very wide variation in opioid prescribing seen across physicians, hospitals, and regions in the United States.^12,21,22^

The 33% relative change in opioid prescribing from 4.0% to 5.3% we observed is consistent with prior literature, although the absolute change in opioid prescribing in our sample was low because of a low baseline rate of prescribing. In an analogous single-center study^10^ on antibiotic prescribing, prescriptions where antibiotics were sometimes or never indicated increased from approximately 40% to 48% throughout the day, a 20% relative increase. In 2 other single-center studies,^23,24^ influenza vaccination rates decreased from 44% to 32% throughout the day, a 27% relative decrease, whereas mammography orders decreased from 63.7% to 47.8%, a 25% relative decrease.

Although variation in treatment patterns across physicians has been well documented, variation in treatment decisions within individual physicians is still little explored. This is surprising given the ubiquity of concern about time pressure and cognitive fatigue as a negative influence on physician burnout, quality of care, and the physician-patient relationship.^9,25,26,27,28,29^ Our results are consistent with prior research^10^ on the association between appointment timing and antibiotic prescribing and at least partially support the theory that physicians prescribe opioids to move on more so than for other nonopioid forms of pain management, such as NSAIDs and physical therapy. We are unable to confirm the exact mechanisms driving these findings because we do not observe physicians’ reasoning or thought process in claims and EHR data alone. However, our findings are consistent with potential mechanisms, including physical or cognitive fatigue, for making effortful decisions such as denying opioid therapy or rushed decision-making when appointments are running late.^30,31,32,33^

Our findings have implications for understanding physician decision-making and quality measurement. First, our results suggest that even within individual physicians, clinical decision-making can be meaningfully influenced by external factors. It is plausible that the patterns we observed extend to other taxing and time-consuming clinical scenarios, such as advanced care planning or starting new medications for chronic disease management. Therefore, over a large population of physicians, the cumulative effect of appointment lateness or long clinical days could have broad clinical outcomes. Interventions to better standardize sensitive treatment decisions, such as shared decision-making tools, could minimize the possibility of external factors influencing decisions such as opioid prescribing. Second, these findings suggest that certain aspects of quality measurement may need to consider appointment timing effects. For example, full-time clinicians may have higher opioid prescribing rates simply because of the effort involved in long clinical days. Sharing individual data on these patterns with physicians could raise awareness of this bias and help them develop approaches such as schedule modifications to lower the burden of taxing or time-consuming decisions late in the day.

Although this study is observational, we sought to minimize selection bias in our results by controlling for considerable baseline variation in opioid prescribing patterns between physicians using physician fixed effects and by using multiple different approaches to examining appointment timing. We also focused on appointments scheduled more than 48 hours in advance to reduce the likelihood that patients chose a time of day related to the severity of their diagnosis. The association between appointment timing and physician prescribing of opioids was consistent across numerous sensitivity analyses. In addition, patterns for the association between timing and nonopioid pain treatments were not present.

### Limitations

This study has several limitations. The results of this observational study should not be interpreted causally, although our findings were robust in several sensitivity analyses addressing selection bias. There could be unobserved reasons why patients more likely to receive opioids may have appointments later in the day, although we observed similar results in our analyses focusing on appointment lateness, which controls for hour of appointment. An additional limitation is that our exposure definition using appointment rank may have included appointments with differing amounts of time pressure depending on the nature of individual physicians’ schedules. Because we relied on data from a convenience sample of primary care physicians who have chosen to purchase the services of athenahealth, our results may not generalize to all primary care physicians in the United States or in developed countries, although in many respects, primary care physicians within the athenahealth network appear to resemble primary care physicians in the United States.^34^ We could not see the full spectrum of each patient’s treatment that they receive from a physician or facility that was not an athenahealth client, which is likely common. Consequently, we may have identified some patients as having no prior opioid use when they were instead receiving opioids from a physician outside the athenahealth system. Our comparison groups of nonopioid therapies also have limitations because patients could receive prescriptions or referrals outside the athenahealth system, for example, with over-the-counter NSAIDs. Also, we could not observe important patient-centered data such as pain severity and effectiveness of prior treatment, so we were unable to assess the clinical appropriateness of opioid prescriptions.

## Conclusions

We found that increases in within-physician opioid prescribing for patients with painful conditions were associated with appointments that occurred later in the day and with appointments that were behind schedule. These results are consistent with the concept that time pressure, through multiple possible mechanisms, may influence physician decision-making. If similar patterns exist in other clinical scenarios, such as managing challenging chronic illness, this phenomenon could have relevance for public health and quality improvement efforts.

## Notes

Supplement. **eAppendix. **Supplemental Methods **eFigure 1.** Distribution of Physicians by Opioid Prescribing Rate **eFigure 2.** Opioid Prescribing by Appointment Order, Odds Ratios **eFigure 3.** Opioid Prescribing by Appointment Lateness, Odds Ratios **eFigure 4.** Antihypertensive and Statin Prescribing, by Appointment Order **eFigure 5.** Antihypertensive and Statin Prescribing, by Appointment Lateness **eTable 1.** Pain Diagnosis ICD-10 Groupings **eTable 2.** Generic Ingredients Included in Opioid and NSAID Prescriptions **eTable 3.** Patient and Appointment Characteristics, Within the athenahealth Sample and the National Ambulatory Care Medical Survey (NAMCS) **eTable 4.** Patient and Appointment Characteristics, by Opioid Prescribing **eTable 5.** Opioid Prescribing by Appointment Order, Full Regression Results **eTable 6.** Patient and Appointment Characteristics, by Appointment Lateness **eTable 7.** Opioid Prescribing by Appointment Lateness, Full Regression Results
