# Supplementary material for: Association of Primary Care Clinic Appointment Time With Opioid Prescribing
Source: JAMA Netw Open. 2019 Aug 30;2(8):e1910373. doi: 10.1001/jamanetworkopen.2019.10373 (PMC6724149; doi:10.1001/jamanetworkopen.2019.10373)
Supplement: Supplement. — eAppendix. Supplemental Methods eFigure 1. Distribution of Physicians by Opioid Prescribing Rate eFigure 2. Opioid Prescribing by Appointment Order, Odds Ratios eFigure 3. Opioid Prescribing by Appointment Lateness, Odds Ratios eFigure 4. Antihypertensive and Statin Prescribing, by Appointment Order eFigure 5. Antihypertensive and Statin Prescribing, by Appointment Lateness eTable 1. Pain Diagnosis ICD-10 Groupings eTable 2. Generic Ingredients Included in Opioid and NSAID Prescriptions eTable 3. Patient and Appointment Characteristics, Within the athenahealth Sample and the National Ambulatory Care Medical Survey (NAMCS) eTable 4. Patient and Appointment Characteristics, by Opioid Prescribing eTable 5. Opioid Prescribing by Appointment Order, Full Regression Results eTable 6. Patient and Appointment Characteristics, by Appointment Lateness eTable 7. Opioid Prescribing by Appointment Lateness, Full Regression Results [file jamanetwopen-2-e1910373-s001.pdf]

Supplementary Online Content

Neprash HT, Barnett ML. Association of primary care clinic appointment time with opioid prescribing. *JAMA Netw Open*. 2019;2(8):e1910373.
doi:10.1001/jamanetworkopen.2019.10373

eAppendix. Supplemental Methods

eFigure 1. Distribution of Physicians by Opioid Prescribing Rate

eFigure 2. Opioid Prescribing by Appointment Order, Odds Ratios

eFigure 3. Opioid Prescribing by Appointment Lateness, Odds Ratios

eFigure 4. Antihypertensive and Statin Prescribing, by Appointment Order

eFigure 5. Antihypertensive and Statin Prescribing, by Appointment Lateness

eTable 1. Pain Diagnosis ICD-10 Groupings

eTable 2. Generic Ingredients Included in Opioid and NSAID Prescriptions

eTable 3. Patient and Appointment Characteristics, Within the athenahealth Sample and the National Ambulatory Care Medical Survey (NAMCS)

eTable 4. Patient and Appointment Characteristics, by Opioid Prescribing

eTable 5. Opioid Prescribing by Appointment Order, Full Regression Results

eTable 6. Patient and Appointment Characteristics, by Appointment Lateness

eTable 7. Opioid Prescribing by Appointment Lateness, Full Regression Results

This supplementary material has been provided by the authors to give readers additional information about their work.

© 2019 Neprash HT et al. *JAMA Network Open.*

eAppendix. Supplemental Methods

Study Sample Definition

To define our study sample, we employed appointment-level, patient-level, and physician-level inclusion criteria. At the appointment level, we limited our sample to the more than 95 percent of appointments scheduled for the four most common durations (10, 15, 20, or 30 minutes) on a weekday (Monday-Friday). Since we relied on time stamps recorded by the EHR during the visit to calculate appointment order and construct other variables, we also restricted our sample based on the quality of each appointments timing data. Specifically, we excluded the small number of appointments with no recorded start or stop time and appointments where the dates for the start and stop time stamps did not match. Finally, we excluded appointments with an encounter starting time stamp more than three hours different from the scheduled visit time, as this likely indicates non-real-time use of the EHR (i.e., delayed appointment documentation).

Our diagnosis-related appointment-level sample restrictions limited the sample to those visits where the provider recorded a diagnosis of pain during the appointment and the patient did not have a cancer diagnosis [140.x-239.x]. We defined pain using the CDC's classification of ICD-9 codes that indicate back or chronic pain. We grouped chronic pain diagnoses into the following categories listed in eTable 1. We limited our sample to appointments with the first occurrence of any diagnosis within a category (e.g., back pain) within the past year, using 2016 claims data.

At the patient level, we excluded pediatric appointments by restricting our sample to visits where the patient was at least 20 years of age. While this exceeds the legal definition of a minor, our data contained age information in 10-year increments. We also limited our analyses to

patients who were opioid naïve at the time of appointment – defined as having no recorded opioid prescription from an athenahealth provider during the past 365 days.

At the provider level, we included physicians (MDs and DOs) with a primary care specialty, including internal medicine, family practice, and general practice. We excluded a small number of physicians who did not prescribe any opioids during our study period, as this likely indicates that they do not use computerized physician order entry (CPOE).

Opioid Prescriptions

We identified opioid prescriptions as any CPOE item listed as a prescription with an enhanced therapeutic class indicating an analgesic narcotic. This included the generic ingredients listed in eTable 2. We excluded opioids designed to treat opioid use disorder or opioid dependence, identified as anything containing the following strings of text: 'methado', ‘dolophine', 'buprenorphine', 'subutex', 'suboxone', ‘buprenex', 'butrans’, 'probuphine', or 'belbuca’. We also excluded the small fraction of prescriptions that were not denominated in milligrams or tablet quantities. We matched opioid prescriptions to appointments using patient identifier, physician identifier, and date.

Non-Opioid Prescriptions

We identified NSAIDs (see eTable 2 for generic ingredients) and statins by searching the enhanced therapeutic class (ETC) label for the ‘%nsaid%’ and ‘%antihyperlipid%’, respectively. We also identified antihypertensive medication prescriptions using the following ETC search terms: ‘%ace i%’, ‘%beta b%’, ‘%calcium c%’, ‘angiotensin%’, ‘%antianginal%’, ‘%diuretic%’, and ‘%vasodilators%’.

© 2019 Neprash HT et al. *JAMA Network Open.*

Physical Therapy Referals

From the universe of all non-prescription orders placed through the EHR, we identified physical therapy referrals as those where the clinical order type name contained the text string '%physical therapy%'.

Chronic Condition Indicators

We defined 27 chronic condition indicators according to the algorithm used by the Chronic Conditions Data Warehouse. When possible, we used the reference period specified in the algorithm. Athenahealth data likely does not include complete claims for all patients, so we anticipate that our measures under capture the presence of chronic conditions within our sample. We rely not on individual indicators, but on a summary measure that counts how many conditions are present.

Regression Specifications

Our main analysis estimated the following linear regression specification:

$$Outcome_{ijt} = \beta_0 + \beta_1 AppointmentTiming_{ijt} + \beta_2 X_{it} + \delta_j + \gamma_t + \epsilon_{ijt}$$

indexing appointment i with physician j at time t. Outcomes of interest were prescribing (opioid, NSAID, antihypertensives, statins) and referral to physical therapy. $AppointmentTiming_{ijt}$ is appointment rank within each physician's workday. X_{it} is a vector of patient and appointment characteristics including patient age category, sex, insurer, chronic condition count, pain type, and scheduled appointment duration. δ_j are physician-practice fixed effects (in reality, these are likely physician fixed effects, but we cannot observe if a physician practices within multiple parent organizations). γ_t are season-year (e.g., Jan-Mar, 2017) fixed effects. Analyses of appointment lateness used a similar equation with appointment start hour as an additional control variable.

© 2019 Neprash HT et al. *JAMA Network Open.*

Sensitivity Analyses

We computed odds ratios for opioid prescribing using appointment-level multivariable logistic regression. The key explanatory variable was an indicator for appointment rank. Other covariates included those in our main model. For computational feasibility, we used physician random effects, rather than fixed effects.

© 2019 Neprash HT et al. *JAMA Network Open.*

eFigure 1. Distribution of Physicians by Opioid Prescribing Rate

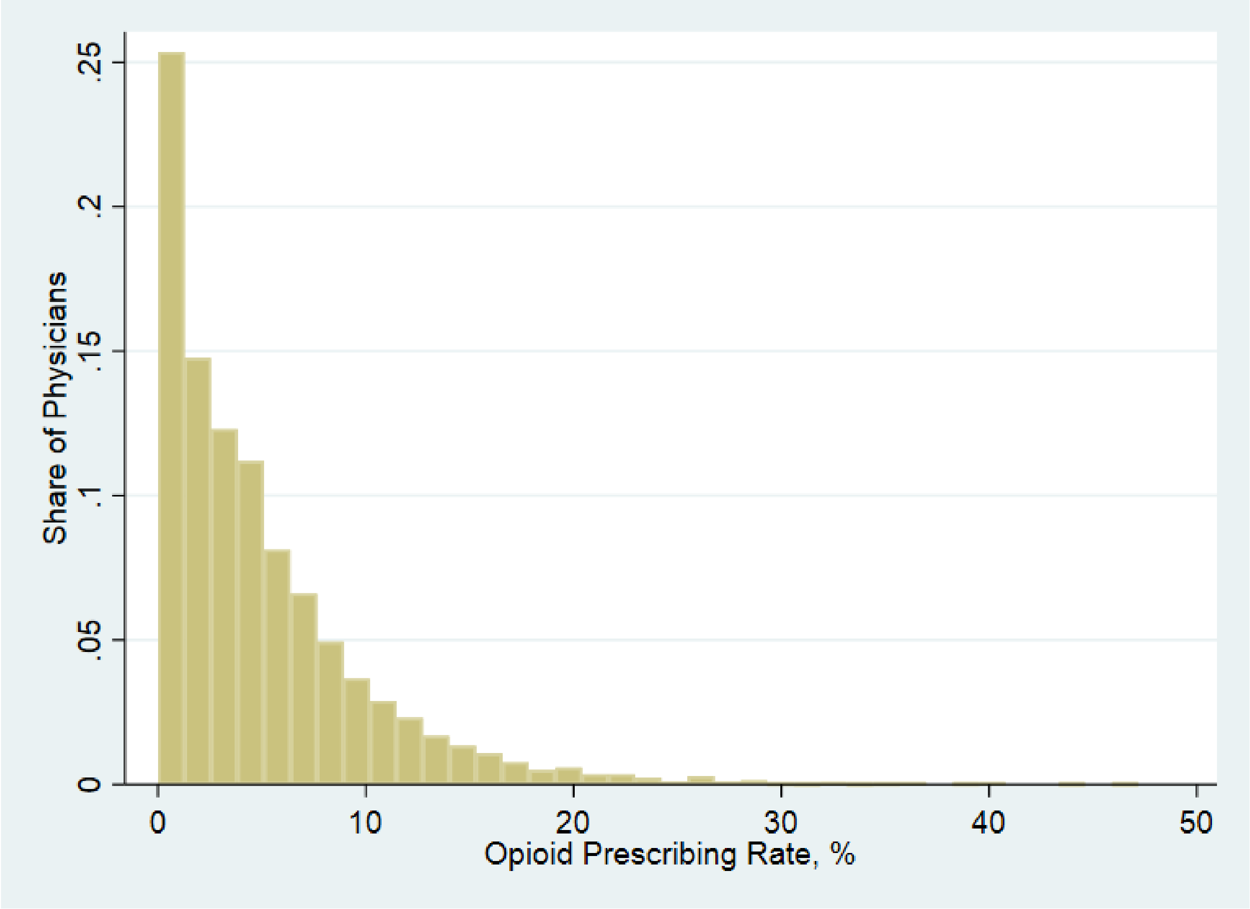

NOTE: This figure plots the distribution of physicians, by their opioid prescribing rate within our main sample. Percentiles are marked on the secondary x-axis.

© 2019 Neprash HT et al. *JAMA Network Open.*

eFigure 2. Opioid Prescribing by Appointment Order, Odds Ratios

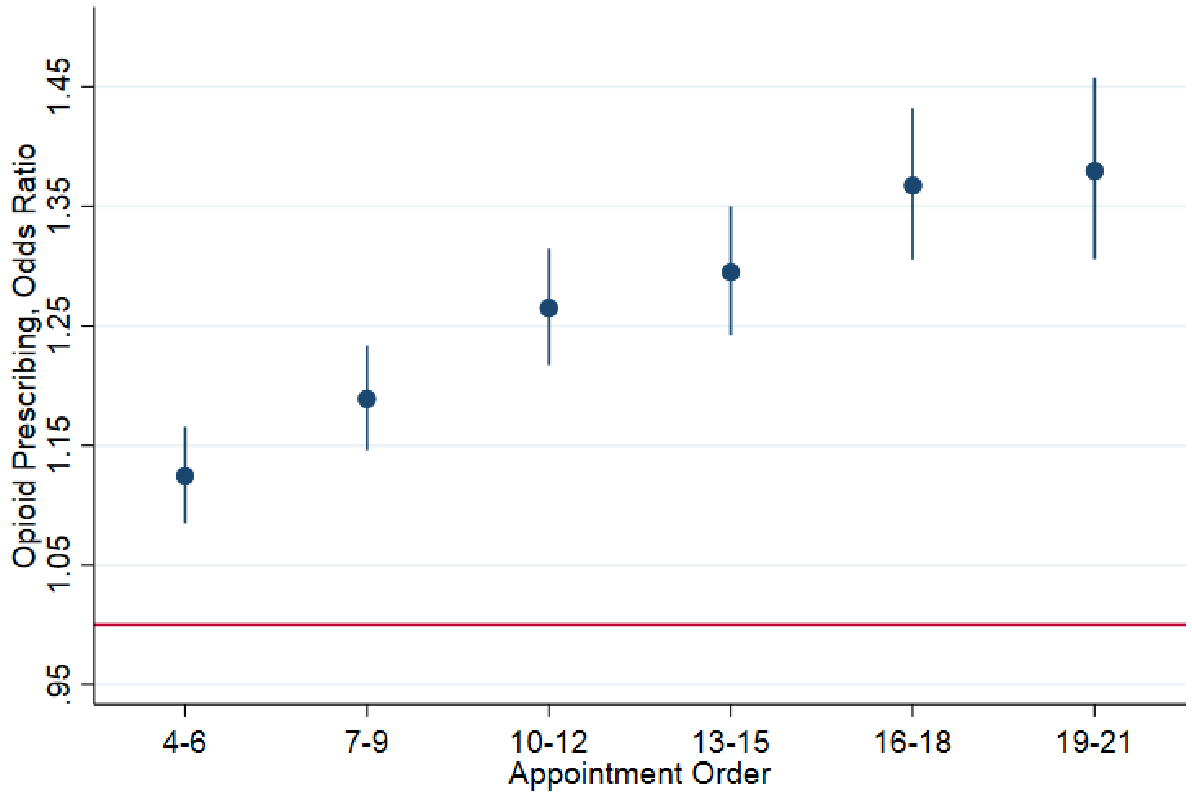

NOTE: Point estimates and confidence intervals were estimated using multivariate logistic regression models with opioid prescribing as the dependent variable. The key covariate was an indicator for appointment order within the day. The model was adjusted as described in Figure 1, with physician random effects, rather than fixed effects.

© 2019 Neprash HT et al. *JAMA Network Open.*

eFigure 3. Opioid Prescribing by Appointment Lateness, Odds Ratios

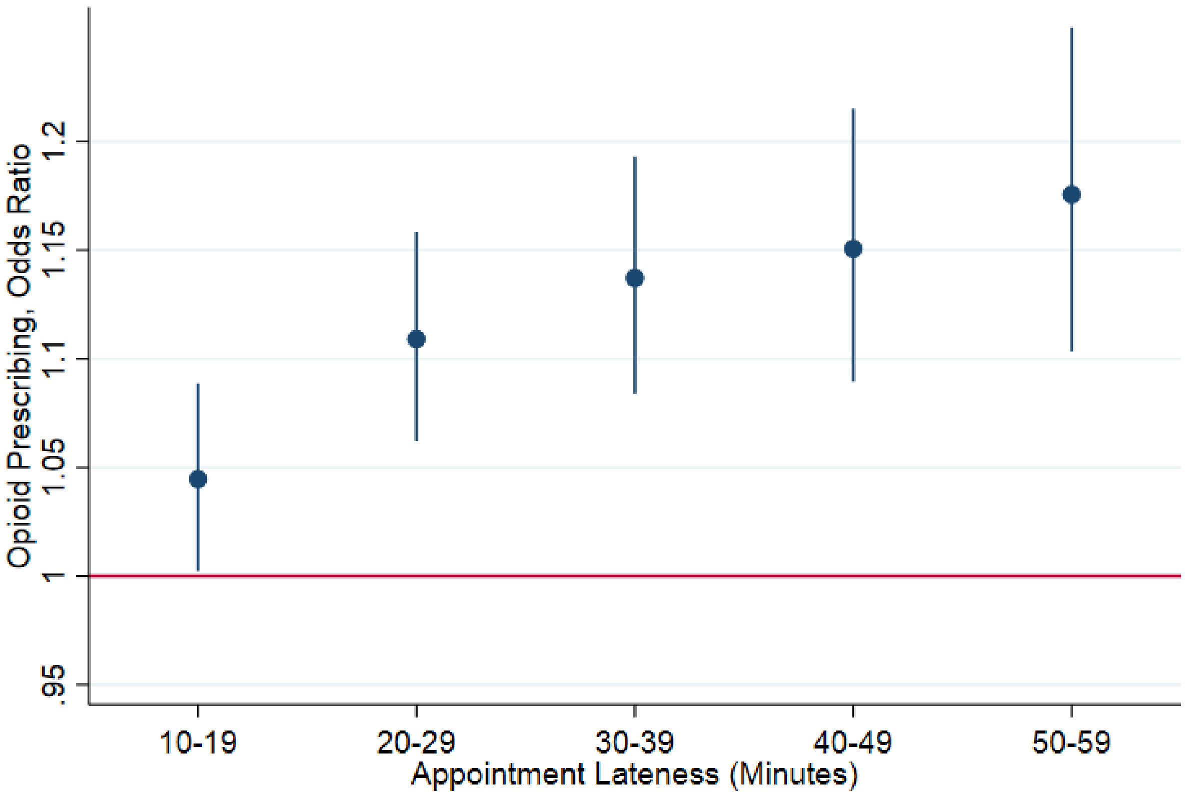

NOTE: Point estimates and confidence intervals were estimated using multivariate logistic regression models with opioid prescribing as the dependent variable. The key covariate was an indicator for appointment lateness. The model was adjusted as described in Figure 1, with physician random effects, rather than fixed effects.

© 2019 Neprash HT et al. *JAMA Network Open.*

eFigure 4. Antihypertensive and Statin Prescribing, by Appointment Order

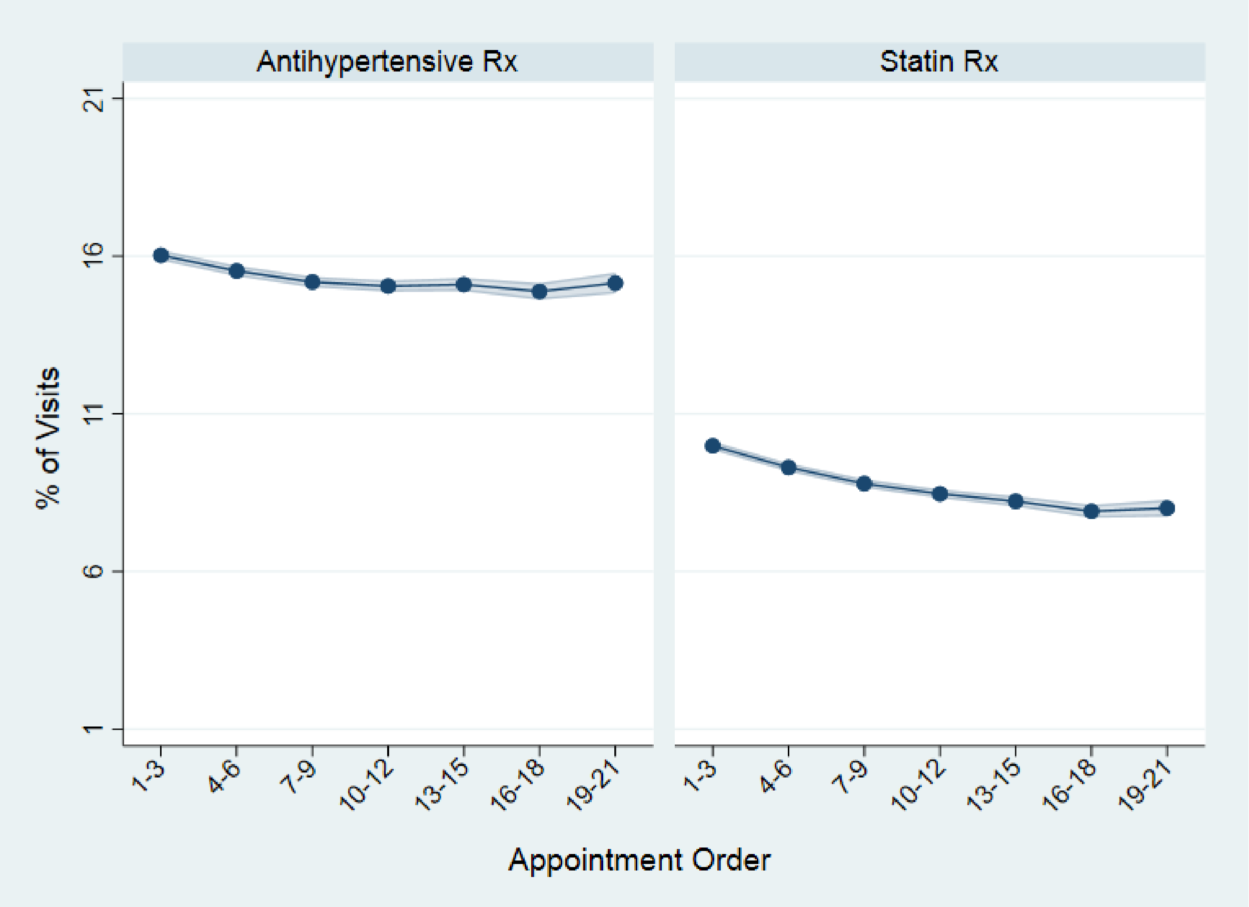

NOTE: Point estimates and confidence intervals were estimated with the use of multivariate linear regression models with antihypertensive and statin prescribing as the dependent variables. The key covariate was an indicator for appointment order. Both models were adjusted as described in Figure 1.

© 2019 Neprash HT et al. *JAMA Network Open.*

eFigure 5. Antihypertensive and Statin Prescribing, by Appointment Lateness

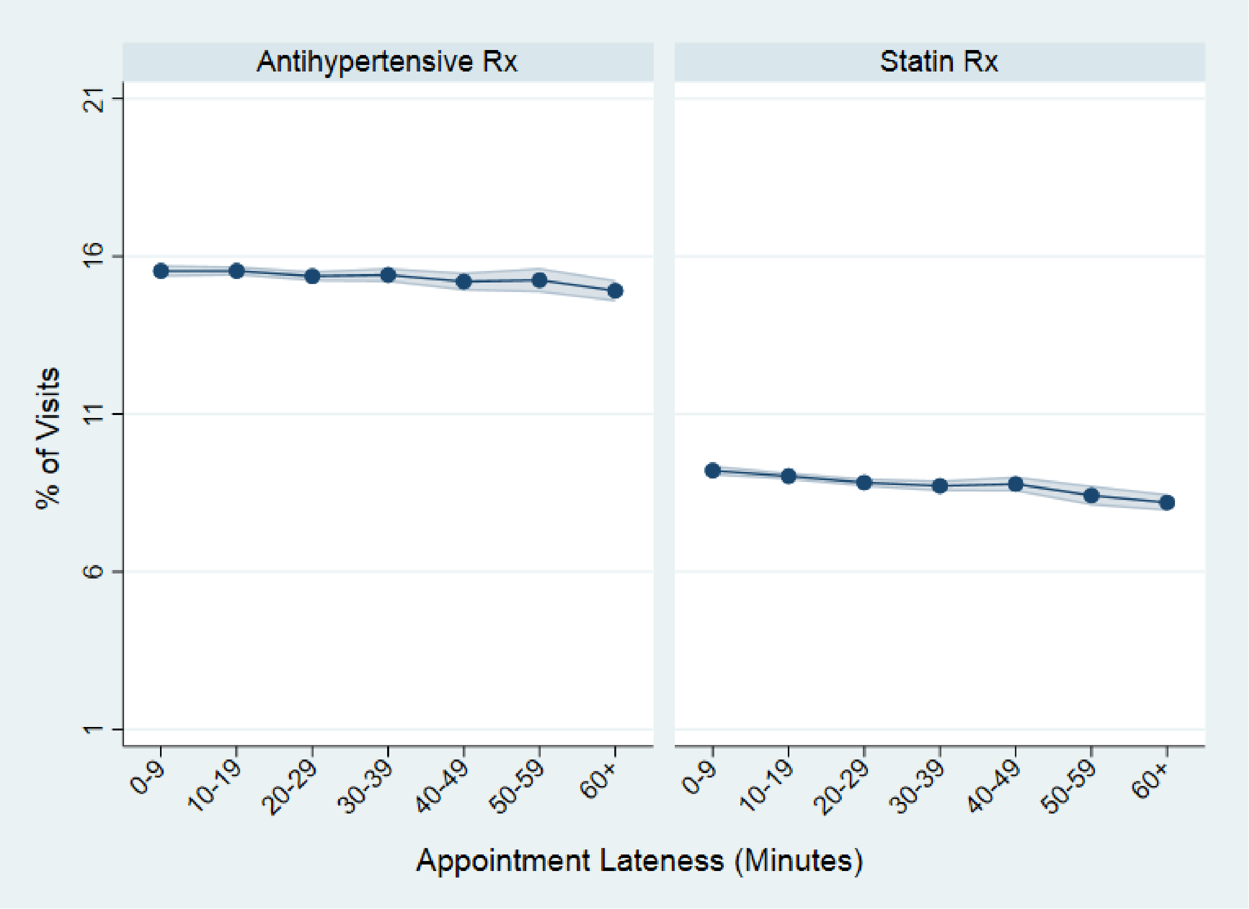

NOTE: Point estimates and confidence intervals were estimated with the use of multivariate linear regression models with antihypertensive and statin prescribing as the dependent variables. The key covariate was an indicator for appointment lateness category. Both models were adjusted as described in Figure 1.

© 2019 Neprash HT et al. *JAMA Network Open.*

eTable 1. Pain Diagnosis ICD-10 Groupings

Back Pain	F45.42, M25.78, M43.2x, M43.6, M43.8X9, M46.4x, M47.x, M48.0x, M48.1x, M48.2x, M48.3x, M48.9, M50.x, M51.x, M53.0, M53.1, M53.2X7, M53.2X8, M53.3, M53.8x, M53.9, M54.0x, M54.11-M54.17, M54.2, M54.3x, M54.4x, M54.5, M54.6, M54.8x, M54.9, M62.830, M96.1, M99.2x, M99.3x, M99.4x, M99.5x, M99.6x, M99.7x, S134XXA, S138XXA, S139XXA, S161XXA, S233XXA, S238XXA, S239XXA, S335XXA, S336XXA, S338XXA, S339XXA
Joint Pain	A18.01, A18.02, A52.16, E08.610, E08.618, E09.610, E09.618, E10.610, E10.618, E11.610, E11.618, E13.610, E13.618, M00.x, M01.Xx, M02.x, M05.x, M06.x, M07.6x, M08.x, M11.x, M12.x, M13.x, M14.x, M15.x, M16.x, M17.x, M18.x, M19.x, M22.x, M23.x, M24.0x, M24.1x, M24.3x, M24.4x, M24.5x, M24.6x, M24.7, M24.8x, M24.9, M25.0x, M25.1x, M25.2x, M25.3x, M25.4x, M25.5x, M25.6x, M25.8x, M25.9, M32.x, M33.x, M34.x, M35.0x, M35.1, M35.2, M35.5, M35.8, M35.9, M36.x, M43.3, M43.4, M43.5Xx, M45.x, M46.0x, M46.1, M46.5x, M46.8x, M46.9x, M48.8x, M49.8x, M53.2X1-M53.2X6, M53.2X9, M79.646, Q68.6, R26.2, R29.4
Musculoskeletal Pain	M20.10, M24.2x, M25.70, M25.71x, M25.72x, M25.73x, M25.74x, M25.75x, M25.76x, M25.77x, M35.4, M35.6, M35.7, M54.10, M54.18, M60.x, M61.x, M62.0x, M62.1x, M62.2x, M62.3, M62.4x, M62.5x, M62.81, M62.82, M62.831, M62.838, M62.84, M62.89, M62.9, M63.x, M65.x, M66.x, M67.x, M70.x, M71.x, M72.x, M75.x, M76.x, M77.x, M79.x, R25.2, R29.898
Migraine	G43.x
Other Pain	G44.209, G89.21, G89.22, G89.28, G89.29, G89.4, M35.3

© 2019 Neprash HT et al. *JAMA Network Open.*

eTable 2. Generic Ingredients Included in Opioid and NSAID Prescriptions

**Drug Class**	**Generic Ingredient**
Opioids	codeine, hydrocodone, hydromorphone, levorphanol, meperidine, morphine, oxycodone, oxymorphone, pentazocine, propoxyphene, tapentadol, tramadol
NSAIDs	celecoxib, diclofenac, etodolac, fenoprofen, flurbiprofen, ibuprofen, indomethacin, ketoprofen, ketorolac, meclofenamic acid, mefenamic acid, meloxicam, nambumetone, naproxen, oxaprozin, piroxicam, sulindac, tolmetin

© 2019 Neprash HT et al. *JAMA Network Open.*

eTable 3. Patient and Appointment Characteristics, Within the athenahealth Sample and the National Ambulatory Care Medical Survey (NAMCS)

		NAMCS (2016)	athenahealth (2017)
Age Category			
	25-44	23.2%	14.5%
	45-64	43.5%	41.7%
	65+	33.3%	43.8%
Sex			
	Female	57.8%	61.4%
*Insurance Status**			
	Commercial	45.5%	46.4%
	Medicare	27.3%	40.8%
	Medicaid	14.1%	10.6%
	Uninsured	2.3%	1.4%
	Other Payer	3.1%	0.7%
Pain Category			
	Back	36.4%	31.6%
	Joint	50.0%	48.1%
	Musculoskeletal	27.2%	28.0%
	Migraine	5.0%	6.4%
	Other	9.5%	4.9%
Chronic Condition (#)		2.0	1.3
*7.7% of insurance status for NAMCS unknown			

NOTE: Percentages may not sum to 100, due to rounding. Both samples include all recorded primary care physician office visits for a painful condition, as defined in eTable 1.

eTable 4. Patient and Appointment Characteristics, by Opioid Prescribing

		Opioid		
		Not Prescribed	Prescribed	P Value
Age Category				
	25-44	14.6%	11.5%	<.001
	45-64	41.7%	41.3%	0.20
	65+	43.7%	47.1%	<.001
Female		61.5%	60.4%	<.001
Insurance Status				<.001
	Commercial	46.9%	36.9%	<.001
	Medicare	40.6%	44.9%	<.001
	Medicaid	6.2	7.8	<.001
	Medicare + Medicaid	4.3%	7.3%	<.001
	Uninsured	1.3%	2.3%	<.001
	Other Payer	0.7%	1.0%	<.001
Pain Category[1]				<.001
	Back	30.7%	49.2%	<.001
	Joint	48.3%	43.6%	<.001
	Musculoskeletal	28.5%	17.3%	<.001
	Migraine	6.5%	4.1%	<.001
	Other	4.6%	10.9%	<.001
Chronic Condition				<.001
	0	33.2%	32.4%	0.00
	1	27.4	26.8	0.01
	2+	39.4	40.8	<.001
Scheduled Duration				<.001
	10 Minutes	7.1%	7.9%	<.001
	15 Minutes	69.5%	71.2%	0.02
	20 Minutes	13.2%	12.7%	<.001
	30 Minutes	10	8	<.001
N=		646,500	31,819	

NOTE: Percentages may not sum to 100, due to rounding. P-values were estimated with the use of Student’s t tests.

© 2019 Neprash HT et al. *JAMA Network Open.*

eTable 5. Opioid Prescribing by Appointment Order, Full Regression Results

	(1)	(2)	(3)
VARIABLES	Unadjusted	Physician Fixed Effects	Main Model
Appointment Order: 1-3	Ref	Ref	Ref
	-	-	-
Appointment Order: 4-6	0.007***	0.006***	0.005***
	(0.001)	(0.001)	(0.001)
Appointment Order: 7-9	0.009***	0.009***	0.007***
	(0.001)	(0.001)	(0.001)
Appointment Order: 10-12	0.012***	0.011***	0.009***
	(0.001)	(0.001)	(0.001)
Appointment Order: 13-15	0.014***	0.012***	0.010***
	(0.001)	(0.001)	(0.001)
Appointment Order: 16-18	0.016***	0.013***	0.013***
	(0.001)	(0.001)	(0.001)
Appointment Order: 19-21	0.017***	0.013***	0.013***
	(0.002)	(0.001)	(0.001)
age: 45-64			0.010***
			(0.001)
age: 65+			0.010***
			(0.001)
Male			0.002***
			(0.001)
Dually Eligible			0.023***
			(0.002)
Medicaid			0.011***
			(0.001)
Medicare			0.008***
			(0.001)
Other Payer			0.014***
			(0.004)
Uninsured			0.029***
			(0.003)
Chronic Condition Count			0.001***
			(0.000)
15 Minutes			0.002
			(0.003)

© 2019 Neprash HT et al. *JAMA Network Open.*

20 Minutes			0.001
			(0.003)
30 Minutes			-0.001
			(0.003)
Joint Pain			0.007***
			(0.001)
Back Pain			0.041***
			(0.001)
Musculoskeletal Pain			-0.005***
			(0.001)
Migraine			0.001
			(0.001)
Other Pain			0.064***
			(0.002)
Apr-Jun, 2017			-0.004***
			(0.001)
Jul-Sept, 2017			-0.008***
			(0.001)
Oct-Dec, 2017			-0.013***
			(0.001)
Observations	678,319	678,319	678,319
R-squared	0.001	0.055	0.068

Note: Point estimates and standard errors were estimated with the use of multivariate linear regression models with opioid prescribing as the dependent variable. Column (2) adjusted for physician fixed effects. Column (3) was adjusted as described in Figure 1. *** p<0.01, ** p<0.05, * p<0.1

© 2019 Neprash HT et al. *JAMA Network Open.*

eTable 6. Patient and Appointment Characteristics, by Appointment Lateness

		Appointment Lateness (Minutes)				
		0-14	15-29	30-60	60+	P Value
Age Category						
	25-44	14.3%	15.3%	15.0%	14.7%	<.001
	45-64	41.7%	42.4%	42.1%	41.6%	<.001
	65+	44.1%	42.3%	42.9%	43.8%	<.001
Female		59.2%	62.2%	63.9%	64.4%	<.001
Insurance Status						
	Commercial	48.5%	48.6%	45.6%	41.0%	<.001
	Medicare	40.9%	39.6%	40.2%	40.6%	<.001
	Medicaid	5.3%	5.9%	6.8%	9.0%	<.001
	Medicare + Medicaid	3.5%	4.0%	5.0%	6.4%	<.001
	Uninsured	1.1%	1.2%	1.6%	2.0%	<.001
	Other Payer	0.6%	0.7%	0.8%	1.0%	<.001
Pain Category[1]						
	Back	30.9%	31.1%	32.3%	34.0%	<.001
	Joint	48.0%	47.7%	47.8%	48.5%	0.004
	Musculoskeletal	28.0%	28.0%	28.0%	28.0%	0.96
	Migraine	6.6%	6.7%	6.3%	5.7%	<.001
	Other	4.5%	4.7%	5.2%	5.9%	<.001
Chronic Condition						
	0	33.8%	34.4%	33.1%	31.5%	<.001
	1	27.2%	27.4%	27.5%	28.0%	0.002
	2+	39.1%	38.2%	39.4%	40.5%	<.001
Scheduled Duration						
	10 Minutes	7.0%	7.1%	7.3%	7.4%	0.002
	15 Minutes	69.4%	69.2%	70.4%	67.9%	<.001
	20 Minutes	13.0%	13.3%	12.8%	15.4%	<.001
	30 Minutes	10.5%	10.3%	9.5%	9.4%	<.001
N=		186,896	196,888	155,916	54,119	<.001

NOTE: Appointment lateness was defined as the difference between observed exam start time (recorded as a time stamp within the EHR) and scheduled start time. Percentages may not sum to 100, due to rounding. P-values are from one-way analyses of variance.

© 2019 Neprash HT et al. *JAMA Network Open.*

eTable 7. Opioid Prescribing by Appointment Lateness, Full Regression Results

	(1)	(2)	(3)
VARIABLES	Unadjusted	Physician Fixed Effects	Main Model
Appointment Lateness: 0-9 Minutes	Ref	Ref	Ref
	-	-	-
Appointment Lateness: 10-19 Minutes	0.002***	0.001	0.001
	(0.001)	(0.001)	(0.001)
Appointment Lateness: 20-29 Minutes	0.007***	0.003***	0.003***
	(0.001)	(0.001)	(0.001)
Appointment Lateness: 30-39 Minutes	0.011***	0.004***	0.004***
	(0.001)	(0.001)	(0.001)
Appointment Lateness: 40-49 Minutes	0.014***	0.005***	0.004***
	(0.001)	(0.001)	(0.001)
Appointment Lateness: 50-59 Minutes	0.017***	0.006***	0.005***
	(0.002)	(0.002)	(0.001)
Appointment Lateness: 60+ Minutes	0.021***	0.009***	0.007***
	(0.002)	(0.001)	(0.001)
age: 45-64			0.010***
			(0.001)
age: 65+			0.010***
			(0.001)
Male			0.002***
			(0.001)
Dually Eligible			0.024***
			(0.002)
Medicaid			0.011***
			(0.002)
Medicare			0.008***
			(0.001)
Other Payer			0.014***
			(0.004)
Uninsured			0.031***
			(0.004)

© 2019 Neprash HT et al. *JAMA Network Open.*

Chronic Condition Count			0.001***
			(0.000)
15 Minutes			0.000
			(0.003)
20 Minutes			-0.001
			(0.003)
30 Minutes			-0.002
			(0.003)
Joint Pain			0.008***
			(0.001)
Back Pain			0.041***
			(0.001)
Musculoskeletal Pain			-0.005***
			(0.001)
Migraine			0.001
			(0.001)
Other Pain			0.065***
			(0.003)
Apr-Jun, 2017			-0.004***
			(0.001)
Jul-Sept, 2017			-0.008***
			(0.001)
Oct-Dec, 2017			-0.014***
			(0.001)
Appointment Start Hour			0.002***
			(0.000)
Observations	593,819	593,819	593,819
R-squared	0.001	0.056	0.070

Note: Point estimates and standard errors were estimated with the use of multivariate linear regression models with opioid prescribing as the dependent variable. Column (2) adjusted for physician fixed effects. Column (3) was adjusted as described in Figure 1. *** p<0.01, ** p<0.05, * p<0.10

© 2019 Neprash HT et al. *JAMA Network Open.*
